# Correction: Iwaniak et al. The Assessment of the Efficacy of Imperatorin in Reducing Overactive Bladder Symptoms. *Int. J. Mol. Sci.* 2023, *24*, 15793

**DOI:** 10.3390/ijms26178268

**Published:** 2025-08-26

**Authors:** Paulina Iwaniak, Piotr Dobrowolski, Jan Wróbel, Tomasz Kluz, Artur Wdowiak, Iwona Bojar, Klaudia Stangel-Wójcikiewicz, Ewa Poleszak, Artur Jakimiuk, Marcin Misiek, Łukasz Zapała, Andrzej Wróbel

**Affiliations:** 1 Department of Experimental and Clinical Pharmacology, Medical University of Lublin, Jaczewskiego 8b, 20-090 Lublin, Poland; 2 Department of Functional Anatomy and Cytobiology, Faculty of Biology and Biotechnology, Maria Curie-Sklodowska University, Akademicka 19, 20-033 Lublin, Poland; 3 Medical Faculty, Medical University of Lublin, 20-093 Lublin, Poland; wrobeljan@onet.eu; 4 Department of Gynecology, Gynecology Oncology and Obstetrics, Institute of Medical Sciences, Medical College of Rzeszow University, Rejtana 16c, 35-959 Rzeszow, Poland; jtkluz@interia.pl; 5 Department of Obstetrics and Gynecology, Faculty of Health Sciences, Medical University of Lublin, Staszica 4-6, 20-081 Lublin, Poland; wdowiakartur@gmail.com; 6 Department of Women’s Health, Institute of Rural Health in Lublin, ul. Jaczewskiego 2, 20-090 Lublin, Poland; iwonabojar75@gmail.com; 7 Department of Gynecology and Oncology, Jagiellonian University Medical College, M. Kopernika 23, 31-501 Kraków, Poland; ksw@cm-uj.krakow.pl; 8 Department of Applied and Social Pharmacy, Laboratory of Preclinical Testing, Medical University of Lublin, Chodźki 1, 20-093 Lublin, Poland; ewapoleszak@umlub.pl; 9 Department of Obstetrics and Gynecology, National Medical Institute of the Ministry of Interior and Administration, Wołoska 137, 02-507 Warsaw, Poland; jakimiuk@yahoo.com; 10 Center for Reproductive Health, Institute of Mother and Child, Kasprzaka 17a, 01-211 Warsaw, Poland; 11 Department of Gynecologic Oncology, Holy Cross Cancer Center, 25-377 Kielce, Poland; marcin.misiek@onkol.kielce.pl; 12 Clinic of General, Oncological and Functional Urology, Medical University of Warsaw, Lindleya 4, 02-005 Warsaw, Poland; lzapala@wum.edu.pl; 13 Second Department of Gynecology, Medical University of Lublin, Jaczewskiego 8, 20-090 Lublin, Poland; wrobelandrzej@yahoo.com

In the published manuscript [1], there was an error regarding the affiliation of Marcin Misiek. In addition to affiliation 11, the updated affiliation should include: Department of Gynecologic Oncology, Holy Cross Cancer Center, 25-377 Kielce, Poland. The authors state that the scientific conclusions are unaffected. This correction was approved by the Academic Editor. The original publication has also been updated.
